# Digital and blended interventions for suicide risk in personality disorders: apps, tele-DBT and text-based supports

**DOI:** 10.1192/j.eurpsy.2026.10677

**Published:** 2026-07-31

**Authors:** D. Jelaga, M. Belous

**Affiliations:** Department of Mental Health, Medical Psychology and Psychotherapy, Nicolae Testemițanu State University of Medicine and Pharmacy, Chisinau, Moldova, Republic of

## Abstract

**Introduction:**

Personality disorders, especially borderline, carry a high suicide burden. Lifetime prevalence from broader-year meta-analyses indicates suicidal ideation ≈80%, attempts ≈52%, and suicide deaths ≈6%. Digital and blended modalities (apps, tele-delivered dialectical behaviour therapy, text-based supports) may expand access; however, effect sizes and safety in PD-only samples remain uncertain.

**Objectives:**

To (1) summarise evidence on suicidal outcomes in PD/BPD; (2) evaluate effectiveness and safety of tele-DBT, apps and text-based supports on ideation, attempts and non-suicidal self-injury; (3) explore moderators by age, setting and intervention features.

**Methods:**

Systematic search of MEDLINE/PubMed, Embase, PsycINFO, Web of Science and Scopus (2020-2025). Adults with clinician-diagnosed PD/BPD; designs: RCTs, longitudinal, quasi-experimental, systematic/narrative syntheses. Outcomes: suicidal ideation/attempts/deaths, NSSI, adverse events, adherence/acceptability. Flow: 1,298 records; 932 after deduplication; 108 full texts; 24 studies included; 84 excluded (no PD subsample/analysis, no suicidal outcomes, usability-only). Samples: ~70–90% female; mean/median age 24–37 years; outpatient/tele-clinic settings. Comparators: treatment-as-usual or in-person DBT where applicable.

**Results:**

BPD lifetime prevalence: ideation 80%, attempts 52%, deaths 6%. Tele-DBT (12-month programme) was associated with NSSI reduction from 80% to 28% (−52 pp; ~65% relative); one hospitalisation after an attempt (~2.6%); dropout 38%; satisfaction 87–97%. A pragmatic RCT (N=580) of an adjunctive digital therapeutic vs treatment-as-usual reported 3-month suicide attempts 2.3% vs 7.6% (−5.3 pp; incidence-rate ratio 0.34). e-DBT via email showed symptom improvements comparable to in-person with completion 44% vs 49%. App-supported/blended approaches yielded small improvements in ideation and mixed effects on NSSI; findings were mostly transdiagnostic with PD subsamples, and features linked to better outcomes included reminders, self-monitoring and skills rehearsal. Text-based supports improved engagement; brief stand-alone online skills training in transdiagnostic samples showed slightly increased self-harm (3.9% vs 3.3%; hazard ratio ≈1.29). Suicide deaths were rarely reported across intervention trials.

**Conclusions:**

Digital/blended interventions, especially tele-DBT and structured adjunctive programmes appear safe and potentially effective complements to standard care for reducing suicidal behaviours in PD/BPD, while effects on NSSI remain mixed. Implementation should prioritise reminders, self-monitoring and guided practice.

**Disclosure of Interest:**

None Declared

